# Comparison of Goal-Directed Hemodynamic Optimization Using Pulmonary Artery Catheter and Transpulmonary Thermodilution in Combined Valve Repair: A Randomized Clinical Trial

**DOI:** 10.1155/2012/821218

**Published:** 2012-04-30

**Authors:** Andrey I. Lenkin, Mikhail Y. Kirov, Vsevolod V. Kuzkov, Konstantin V. Paromov, Alexey A. Smetkin, Mons Lie, Lars J. Bjertnæs

**Affiliations:** ^1^Cardiosurgical Intensive Care Unit, City Hospital No. 1, Suvorov Street 1, Arkhangelsk 163001, Russia; ^2^Department of Anesthesiology and Intensive Care Medicine, Northern State Medical University, Troitsky Avenue 51, Arkhangelsk 163000, Russia; ^3^Department of Anesthesiology, University Hospital of North Norway, Tromsø, Norway; ^4^Department of Clinical Medicine (Anesthesiology), Faculty of Medicine, University of Tromsø, 9037 Tromsø, Norway; ^5^Office for International Cooperation, Oslo University Hospital, Kirkeveien 166, 0407 Oslo, Norway

## Abstract

Our aim was to compare the effects of goal-directed therapy guided either by pulmonary artery catheter (PAC) or by transpulmonary thermodilution (TTD) combined with monitoring of oxygen transport on perioperative hemodynamics and outcome after complex elective valve surgery. *Measurements and Main Results*. Forty patients were randomized into two equal groups: a PAC group and a TTD group. In the PAC group, therapy was guided by mean arterial pressure (MAP), cardiac index (CI) and pulmonary artery occlusion pressure (PAOP), whereas in the TTD group we additionally used global end-diastolic volume index (GEDVI), extravascular lung water index (EVLWI), and oxygen delivery index (DO_2_I). We observed a gradual increase in GEDVI, whereas EVLWI and PAOP decreased by 20–30% postoperatively (*P* < 0.05). The TTD group received 20% more fluid accompanied by increased stroke volume index and DO_2_I by 15–20% compared to the PAC group (*P* < 0.05). Duration of mechanical ventilation was increased by 5.2 hrs in the PAC group (*P* = 0.04). *Conclusions*. As compared to the PAC-guided algorithm, goal-directed therapy based on transpulmonary thermodilution and oxygen transport increases the volume of fluid therapy, improves hemodynamics and DO_2_I, and reduces the duration of respiratory support after complex valve surgery.

## 1. Introduction

Valve repair and replacement is a rapidly progressing and challenging type of cardiac surgery [1–3]. The outcome of valve surgery is influenced by a variety of factors including age and the general condition of the patient, preoperative severity of heart dysfunction, myocardial ischemia, and duration of cardiopulmonary bypass (CPB) [4, 5]. The latter may induce systemic inflammatory response syndrome (SIRS) and lead to multiorgan dysfunction syndrome (MODS) [6–10].

Several therapeutic approaches have been used to alleviate CPB-induced SIRS and MODS including goal-directed hemodynamic optimization [11]. Thus, complex monitoring could increase the efficacy of these therapies. Recently, so-called “less invasive” techniques for measurement of cardiac output (CO) have been implemented as a useful adjunct or even alternative to the hemodynamic monitoring by means of the pulmonary artery catheter (PAC). Among these various techniques, transpulmonary thermodilution, allowing measurement of volumetric parameters and subsequent continuous, “beat-to-beat” CO-computation based on pulse contour analysis, has proved to be a valuable monitoring tool both in coronary surgery and heart failure [12–15]. However, its potential advantage in heart valve surgery in comparison with pressure-oriented hemodynamic monitoring, which is still widely used, has not been elucidated. This is especially interesting when taking into account that, before repair, valve diseases can distort the thermodilution curves, and thus, the results of the measurements.

Severe SIRS and MODS triggered by major cardiosurgical intervention and/or CPB can also disturb the oxygen transport. Hence, continuous measurement of either central venous (ScvO_2_) or mixed venous (SvO_2_) oxygen saturation may be a valuable adjunct to routine hemodynamic monitoring, which allows the determination of oxygen delivery and improves the outcome of several categories of critically ill patients [16, 17]. Recently introduced in clinical practice, the combination of continuous monitoring of CO and oxygen transport seems to be an attractive tool for displaying a “global hemodynamic view” and subsequent goal-directed perioperative optimization [18]. These algorithms have demonstrated their feasibility in both on-pump [19] and off-pump [15] coronary artery bypass grafting, but require further investigation in valve repair and replacement.

Therefore, the aim of this study was to evaluate the effect of treatment algorithms guided either by PAC or by transpulmonary thermodilution combined with monitoring of oxygen transport on perioperative hemodynamic management and outcome after complex valve surgery.

## 2. Materials and Methods

The study was approved by the Ethics Committee of Northern State Medical University, Arkhangelsk, Russia, in full compliance with the ethical standards as proclaimed by the Helsinki Declaration. Written informed consent was obtained from all patients or legal surrogate.

 Forty-three adult patients scheduled for elective replacement/repair of two and more valves were enrolled into the single-centre study performed in an 850-bed university hospital during the period from March 2008 to June 2010. All operations were performed by the same surgical team. The inclusion criteria were age >18 years, presence of moderate or severe valve regurgitation and/or stenosis, and scheduled repair and/or replacement of two or more cardiac valves requiring CPB. The patients were excluded from the study if they had stenosis of coronary arteries requiring simultaneous coronary bypass grafting, extreme obesity (body mass index >40 kg m^−1^), or participation in other investigations. Before the procedure, all patients were examined according to a standard protocol; risk of surgery was evaluated using the EuroSCORE system [20].

### 2.1. Clinical Protocol

**Figure 1:**
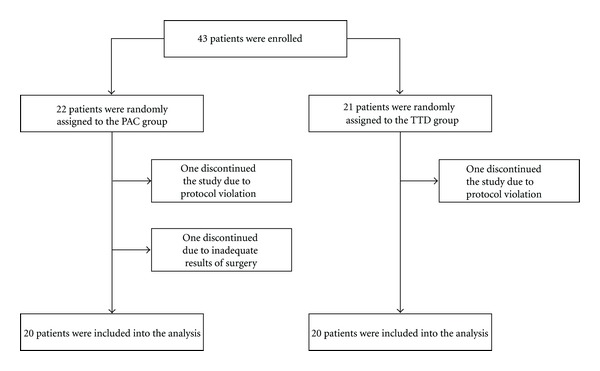
Flow diagram detailing the conduct of the study. PAC: pulmonary arterial catheter; TTD: transpulmonary thermodilution.

**Figure 2:**
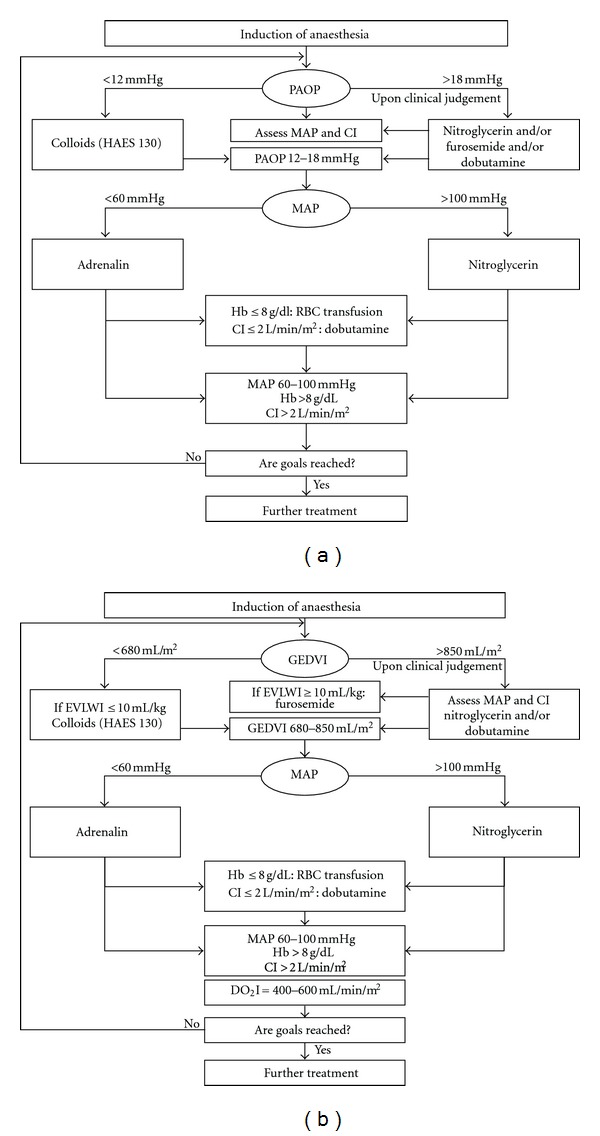
The algorithms of goal-directed hemodynamic optimization: (a) the PAC group, (b) the transpulmonary thermodilution (TTD) group. CPB: cardiopulmonary bypass; MAP: mean arterial pressure; PAOP: pulmonary artery occlusion pressure; CI: cardiac index; GEDVI: global end-diastolic volume index; EVLWI: extravascular lung water index; ScvO_2_: central venous oxygen saturation; DO_2_I: oxygen delivery index; Hb: hemoglobin concentration; RBC: red blood cells; HAES: hydroxyaethyl starch.

On the day of surgery, patients were randomized into two groups using unmarked, sealed envelopes. Three patients were excluded from the analysis (Figure 1): two due to protocol violation (inability to reach study goals postoperatively caused in one case by massive blood loss and in another case—by PAC malfunction) and one due to inadequate surgical correction diagnosed by intraoperative transesophageal echocardiography (TEE). The hemodynamic optimization in the PAC group (*n* = 20) was targeted using parameters provided by PAC including pulmonary arterial occlusion pressure (PAOP) and cardiac index (CI) (LifeScope monitor, Nihon Kohden, Japan) (Figure 2(a)) (In cases of a PAOP < 12 mm Hg, a 500 mL bolus of 6% hydroxyaethyl starch 130/0.42 (Venofundin, B | Braun) was infused over 30 minutes aiming at a PAOP within the range of 12–18 mm Hg. The bolus infusion could be repeated once. If PAOP exceeded 18 mm Hg, nitroglycerin and/or furosemide and/or dobutamine were used on clinical judgment. If MAP was <60 mm Hg, an epinephrine infusion was started at 0.05 *μ*g kg^−1^ min^−1^ with the option to increase the dose in 0.05 *μ*g kg^−1^ min^−1^ increments, if required. In case of hypertension (MAP > 100 mm Hg), nitroglycerin infusion was administered in the dose range of 0.5–3.0 *μ*g kg^−1^ min^−1^. A transfusion trigger was Hb < 8 g dL^−1^. Heart failure and low cardiac output syndrome (CI < 2.0 L min^−1^ m^−2^) required a dobutamine infusion starting at 3.0 *μ*g kg^−1^ min^−1^. Central venous oxygen saturation (ScvO_2_) was maintained >60%). In the group of transpulmonary thermodilution, the TTD group (*n* = 20), hemodynamics was managed using transpulmonary thermodilution including CI, global end-diastolic volume index (GEDVI), extravascular lung water index (EVLWI), and oxygen delivery index (DO_2_I) as measured with the PiCCO_2_ monitor (Pulsion Medical Systems, Munich, Germany) (Figure 2(b)) (In cases where GEDVI < 680 mm^−2^ and EVLWI < 10 mL kg^−1^, a 500 mL bolus of 6% hydroxyaethyl starch 130/0.42 was infused over 30 minutes aiming at a GEDVI within the range of 680–850 mL m^−2^. The bolus infusion could be repeated. If GEDVI exceeded 850 mL kg^−1^, nitroglycerin and/or furosemide and/or dobutamine were given on clinical judgement. In case of pulmonary edema (EVLWI > 10 mL kg^−1^), we used intravenous administration of furosemide at a dose of 20 mg. If MAP was < 60 mm Hg, epinephrine infusion was started at 0.05 *μ*g kg min^−1^ with an optional increment in dosage of 0.05 *μ*g kg^−1^ min^−1^. In cases of hypertension (MAP > 100 mm Hg), nitroglycerin infusion was given at a dose of 0.5–3.0 *μ*g kg^−1^ min^−1^. A transfusion trigger was Hb < 8.0 g dL^−1^. Heart failure and low cardiac output syndrome (CI < 2.0 L min^−1^ m^−2^) were treated with a dobutamine infusion starting at 3.0 *μ*g kg^−1^ min^−1^ aimed at maintaining DO_2_I in the range of 400–600 mL min^−1^ m^−2^. ScvO_2_ was maintained >60%). Mean arterial pressure (MAP), heart rate (HR), and hemoglobin concentration (Hb) were included into both the PAC- and the TTD-driven protocols. In both groups, ScvO_2_ was maintained >60%. The algorithms for perioperative goal-directed therapy are depicted in Figure 2.

### 2.2. Anesthesia, Surgery, and Postoperative Care

All patients received standard premedication with diazepam. After arrival to the operation theatre, a femoral artery was catheterized either with standard 18G catheter (Arteriofix, B | Braun, Germany) in the PAC-group or with a 5F thermodilution catheter (PV2015L20 PULSOCATH, Pulsion Medical Systems) in the TTD group. After induction of anesthesia in the PAC group, a central venous introducer (Intradyn 8F, B | Braun) was inserted into the right internal jugular vein followed by a PAC (7.5F, Corodyn, B | Braun). The position of PAC and the adequacy of valve repair were verified by TEE (Acuson Cypress, Siemens, Germany) performed after CPB. In the TTD group, a triple-lumen central venous catheter (Certofix, B | Braun) and a fibre-optic probe (PV 2022–37, Pulsion Medical Systems) were inserted via the right jugular vein for continuous oxygen transport monitoring. Central venous pressure (CVP) was measured using either the venous port of the PAC or the middle port of the triple-lumen catheter in the PAC and the TTD groups, respectively.

Induction of anesthesia was performed with midazolam 0.07 mg kg^−1^, propofol 1.0 mg kg^−1^ and fentanyl 5–7 *μ*g kg^−1^ in both groups. Anesthesia was maintained by continuous infusion of propofol (3–5 mg kg^−1 ^hr^−1^) and fentanyl (4-5 *μ*g kg^−1 ^hr^−1^). Muscular paralysis for tracheal intubation was achieved by pipecuronium bromide 0.1 mg kg^−1^ and maintained with repeated doses of pipecuronium 0.015 mg kg^−1 ^hr^−1^ during operation. After intubation, volume-controlled mechanical ventilation (Fabius GS, Dräger, Germany) was provided with FiO_2_ 0.5, tidal volume 7-8 mL kg^−1^, positive end-expiratory pressure (PEEP) 5 cm H_2_O, and respiratory rate of 12–14 min^−1^. For postoperative mechanical ventilation, we used Evita 4 (Dräger, Germany), maintaining a tidal volume of 7-8 mL kg^−1^ and a PEEP of 5 cm H_2_O.

Cardiopulmonary bypass was performed in nonpulsatile mode with perfusion index of 3.0 L min^−1 ^m^−2^ using a standard roller-pump CPB-machine (Jostra HL 20, Maquet, Sweden). The priming of the reservoir was similar in both groups: 1000 mL Ringer's solution and 500 mL Gelofusine (B | Braun). For cardiac arrest and myocardial protection, we infused ice-cold (4–6°C) cardioplegic solution (Custodiol, Dr. F. Koehler Chemie GmbH, Germany) antegradely at an initial dose of 20 mL/kg. Restoration of cardiac function was either spontaneous or facilitated by means of an epicardial pacemaker. Weaning from CPB was performed in a stepwise manner. In case of heart failure diagnosed as CI below 2.0 L min^−1 ^m^−2^, we used dobutamine and/or epinephrine. Fluid replacement included crystalloid solutions (Sterofundin Iso/G5, B | Braun) with an initial infusion rate 6-7 mL kg^−1 ^hr^−1^ prior to and during anesthesia and 2-3 mL kg^−1 ^hr^−1^ postoperatively.

### 2.3. Measurements

In both groups, hemodynamic parameters as well as arterial and central venous blood gases, arterial hemoglobin, and lactate and glucose concentrations using ABL800Flex (Radiometer, Denmark) were evaluated after induction of anesthesia, at the end of surgery, and at 2, 6, 12, 18, and 24 hrs postoperatively. These perioperative stages were selected for goal-directed hemodynamic adjustments. In addition, plasma samples were taken before surgery and at 24 hrs postoperatively for the determination of probrain natriuretic peptide (NT-proBNP).

During the study, we evaluated perioperative fluid therapy, fluid balance, and inotrope/vasoactive support. The severity of postoperative MODS was estimated using the SOFA score [21]. For assessment of clinical outcome, we used duration of postoperative mechanical ventilation as the primary end-point and the length of ICU and hospital stay, and the mortality rate at Day 28 as the secondary end-points. The clinician responsible for the weaning from ventilation, ICU stay, and patient discharge was not involved in the study.

Criteria for termination of postoperative respiratory support were the following: a cooperative patient; adequate muscular tone; SpO_2_ > 95% with FiO_2_ 0.5; PaCO_2_ <45 mm Hg; postoperative bleeding rate <50 mL hr^−1^; stable hemodynamics without inotrope/vasopressor support; body temperature of >35°C. Temporary pacing was not regarded as a contraindication for tracheal extubation.

Length of ICU stay was registered when the patient's condition met the following “fit for discharge” criteria: fully oriented, SaO_2_ > 90% on room air, no episodes of severe arrhythmias, bleeding <50 mL hr^−1^, diuresis >0.5 mL kg^−1 ^hr^−1^, no need for inotrope/vasopressor support, and no signs of ischemia on ECG.

The patients were discharged from hospital when they satisfied the following criteria: hemodynamic stability, independence of ambulation and feeding, afebrile with no obvious infections, normal voiding and bowel movements, pain control on oral medications, and exercise tolerance.

### 2.4. Statistical Analysis

The SPSS 15.0 software package was used for statistical analysis. Calculation of sample size was based on initial observations (10 cases in each group) and the hypothesis that TTD will shorten the time of postoperative mechanical ventilation by 5 hrs compared with the PAC group. In order to find a statistically significant difference with *α* of 0.05 and power of 0.8, a sample size of 20 patients in each group proved to be sufficient. Data were checked for normal distribution by means of the Kolmogorov-Smirnov's test. Values are presented as mean ± standard deviation (SD) or median (25th–75th percentiles) for parametrically or nonparametrically distributed variables, respectively. In compliance with the distribution of data, Student's *t*-test or Mann-Whitney's *U* test were used for comparisons between groups. Intragroup comparisons were performed using test of contrasts. Discrete data were analyzed by two-sided *χ* ^2^-test or Fisher's exact test. For all tests, a *P* value < 0.05 was considered as significant.

## 3. Results

As shown in Table 1, we found no intergroup differences regarding demographic data, risk of surgery and severity of chronic illnesses, severity of heart failure, preoperative ejection fraction, durations of surgery, aortic cross-clamping, and CPB.

### 3.1. Hemodynamic Parameters

Table 2 demonstrates the changes in hemodynamics. In both groups, CVP rose at the end of surgery. Postoperatively, CVP declined transiently in the PAC group (*P* < 0.05) but returned to the baseline values by 24 hrs. In contrast, in the TTD group, CVP exceeded the corresponding values of the PAC group at 6 and 18 hrs (*P* < 0.05). In the TTD group, we observed a gradual postoperative increase in GEDVI and stroke volume variations (SVV) starting from 12 and 18 hrs, respectively, whereas EVLWI decreased by 20–30% (*P* < 0.05). In the PAC group, PAOP decreased significantly after operation.

By the end of intervention, MAP and SVRI were higher in the TTD group (Table 2; *P* < 0.05). Postoperatively, MAP and HR rose in both groups whereas SVRI decreased until 6 hrs compared with the preoperative values (*P* < 0.05). At 12 hrs, SVRI increased in the PAC group (*P* = 0.03), but decreased beyond 12 hrs postoperatively in the TTD group.

**Figure 3:**
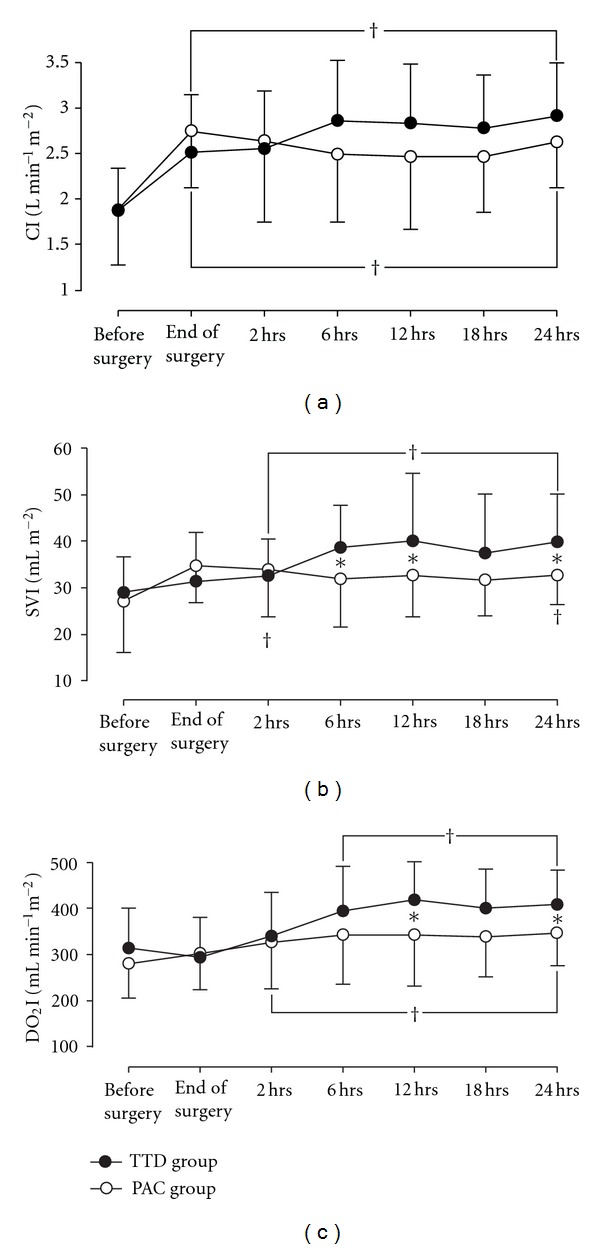
Changes in cardiac index, stroke volume index and oxygen delivery in the study groups. CI: cardiac index; SVI: stroke volume index; DO_2_I: oxygen delivery index. *P < 0.05 between the groups; ^†^ *P* < 0.05 within the group compared with the preoperative value. Data are presented as mean ± SD.

As shown in Figure 3, CI rose postoperatively by 55% in the TTD group and by 41% in the PAC group without intergroup difference. In parallel, SVI and DO_2_I increased after the operation in both groups. However, from 6 hrs postoperatively SVI and DO_2_I were higher by 15–20% in the TTD group (*P* < 0.05).

### 3.2. Oxygenation/Laboratory Parameters

Oxygenation and other laboratory data are shown in Table 3. Oxygenation ratio (PaO_2_/FiO_2_) did not differ significantly between the groups. At the end of surgery, PaO_2_/FiO_2_ decreased transiently in the TTD group, whereas ScvO_2_ increased in comparison with the preoperative values in the PAC group (*P* < 0.05). At 12 hrs, ScvO_2_ was higher in the PAC group (*P* = 0.012). After the intervention, pH decreased transiently in parallel with a rise in plasma lactate and a decline in Hb in both groups (*P* < 0.05) without intergroup differences. Base excess (BE) and PaCO_2_ did not differ between the groups.

Postoperatively, we observed hyperglycemia, which was more pronounced in the PAC group but without significant intergroup difference (Table 3). The plasma concentrations of NT-proBNP rose postoperatively by 1045 pg mL^−1^ and 1315 pg mL^−1^ in the TTD and the PAC groups, respectively (*P* > 0.05). Preoperative serum creatinine concentrations were 0.08 ± 0.02 mmol L^−1^ and 0.09 ± 0.03 mmol L^−1^ in the TTD and the PAC groups, respectively. At 24 hrs after surgery, there was a trend towards increased creatinine values in the PAC group (0.148 ± 0.02 mmol L^−1^ versus 0.125 ± 0.03 mmol L^−1^) in the TTD group (*P* = 0.08).

### 3.3. Clinical Characteristics and Outcomes

The clinical characteristics and outcomes are presented in Table 4.

Although the volume of crystalloids administered during surgery did not differ significantly between the groups, the TTD group received 24% more crystalloids and a threefold more colloids postoperatively (*P* < 0.05). The total volume of postoperative fluid therapy in this group exceeded that of the PAC group by 20% (*P* = 0.01). The incidence of colloid administration and the postoperative fluid balance tended to be higher in the TTD group; by contrast, the incidence and duration of inotropic/vasopressor support in this group demonstrated a trend towards lower doses as compared to the PAC-monitored patients. The incidence of diuretic administration, postoperative diuresis, blood loss and transfusion requirements, and the SOFA score at 24 hrs did not differ between the groups. The rate of pericardial pacing was similar: 70% and 60% in the PAC group and the TTD group, respectively.

The requirement for renal replacement therapy was also similar (one patient in each group). One patient in each group presented with a postoperative stroke. There was no wound infection in the studied patient population.

Duration of postoperative respiratory support increased by 36% in the PAC group (Table 4, *P* = 0.04). However, the duration of ICU stay and hospitalization did not differ. All the patients included in the study survived at Day 28.

## 4. Discussion

The study demonstrates that transpulmonary thermodilution combined with continuous monitoring of oxygen delivery may be used for detection of disorders in hemodynamics and oxygen transport that might influence the perioperative therapy after complex valve surgery.

Complex valve repair results in significant changes in preload. In this study, we found an increase in CVP after CPB in both groups, which is typical for these cardiac interventions [22]. In the TTD group, GEDVI rose after surgery in parallel with increased fluid therapy, whereas EVLWI declined. This finding can be explained by inclusion of colloids according to the treatment algorithm and by the rise in myocardial performance following valve repair. Postoperatively, the patients in the PAC group displayed decreases in the CVP and PAOP values. The reduction in these preload parameters may be caused by several mechanisms: by discontinuation of mechanical ventilation with PEEP and restoration of spontaneous breathing; for the second, from increased heart performance, and finally, from the relatively restrictive fluid regimen in the PAC group. The increase of SVV that we observed in patients of the TTD group at the end of the first postoperative day may be explained mainly by cessation of respiratory support. These results correspond with other studies of goal-directed therapy in cardiac surgery [12, 19, 23, 24].

At the end of surgery, we found lower MAP and SVRI values in the PAC group. Systemic vasodilatation can be explained by the CPB-induced SIRS that might be attenuated by the TTD-driven fluid therapy including colloids [11, 25]. In contrast to the TTD group, the patients of the PAC group presented with systemic vasoconstriction postoperatively, as evidenced by the increase in SVRI, which we interpret as a compensatory mechanism counteracting the reduced blood volume [26].

In addition to the changes in afterload, both groups had increased postoperative heart rate and myocardial contractility that is confirmed by an increase in CI and SVI. These changes can be caused by correction of the valvular malfunctions, restoration of myocardial function and hemodilution in parallel with fluid therapy [27]. Despite the transient perioperative changes in arterial and central venous oxygenation, we observed an increase in oxygen delivery in parallel with regress of metabolic acidosis at 24 hrs postoperatively in both groups. These results confirm the efficacy of the goal-directed hemodynamic optimization. Therapy that increased oxygen transport attenuates the surgical stress and the hypoperfusion following combined CPB and valve repair [28]. In our investigation, this stress was manifested by hyperglycemia, a rise in NT-proBNP, and increase in plasma lactate in both groups. Similar findings have been described by other authors who assessed the effects of CPB and combined valve surgery [29, 30].

The preload optimization following valve repair in the TTD group might have contributed to an increase in heart performance with higher SVI compared with the PAC group. Similar results were obtained by Hofer et al. in a general ICU population [31] and by Brock et al. in patients undergoing cardiac surgery [32]. As a result of goal-directed therapy, the patients in the TTD group received more crystalloids and colloids and tended to receive less inotropic and vasopressor agents postoperatively. In cardiosurgical patients, similar results have been reported [19, 33]. Correction of hypovolaemia and cardiac output according to the study algorithm resulted in a better oxygen delivery and reduced the duration of respiratory support in the TTD group. These findings are consistent with beneficial effects of goal-directed therapy both in coronary and general surgery patients [15, 18, 19].

The observed intergroup differences might not result solely from the net volume of fluids but also from the accuracy of hemodynamic parameters used for preload assessment. Indeed, PAOP has been demonstrated to have a limitation as a preload marker [34]. In contrast, GEDVI is a more reliable marker of preload indicating the filling volume of all heart chambers, while PAOP barely reflects filling pressure of the left atrium [35].

Perioperative goal-directed therapy should be early, adequate, and individualized. Maintaining “supranormal” cardiac output and oxygen delivery does not improve the clinical outcome [36], thus we targeted to keep DO_2_I values within the range of 400–600 mL min^−1 ^m^−2^. Although one of the aims of our treatment algorithms in both groups was to maintain CI > 2.0 L min^−1 ^m^−2^, we did not reach mean DO_2_I values > 400 mL min m^−2^ in the PAC group. Interestingly, despite lower oxygen delivery, mean ScvO_2 _at 12 hrs was higher in the PAC group, which might indicate decreased oxygen consumption. Thus, although CI and ScvO_2_ are important determinants of oxygen transport in high-risk patients, they should be accompanied by assessment of DO_2_I for the most efficient guidance of postoperative care. Moreover, some conditions such as severe pulmonary hypertension might require simultaneous measurement of both volumetric parameters and pulmonary arterial pressures, using either PAC catheter or echocardiography for optimization of the hemodynamic management.

Better oxygen transport might influence organ function and improve clinical outcome. In our study, the PAC group tended to present with increased plasma creatinine concentrations postoperatively. This group received less fluid, which possibly contributed to hypoperfusion and impaired renal function [37]. Other investigators have demonstrated that perioperative goal-directed therapy may have a protective effect on organ function, reducing the number of complications and even decreasing mortality, especially in high-risk patients [16, 18, 38].

This study has several limitations related to the differences in study algorithms. Firstly, we did not measure PAOP in the TTD group or GEDVI in the PAC group. The reason was that the possibility for the attending physician to evaluate the volumetric parameters in the PAC group and the PAC-derived variables in the TTD group could have influenced the choice of fluid therapy. Secondly, in the PAC group, in contrast to the TTD group, DO_2_I was determined intermittently and was not included in the algorithm of goal-directed therapy. However, although the oxygen transport in the TTD group was monitored continuously, it required calibration with discrete measurement of blood gases at the same time points like in the PAC group. Moreover, this single-centre study has a limited number of observations and was not powered for demonstrating the reduction in ICU and hospital stay in the TTD group.

## 5. Conclusions

As compared to a PAC-guided treatment algorithm, goal-directed therapy based on transpulmonary thermodilution combined with monitoring of oxygen transport changes the strategy of fluid management, which in turn, improves hemodynamics and oxygen delivery and reduces the duration of postoperative respiratory support after complex valve surgery.

## Figures and Tables

**Table 1:** Pre- and intraoperative characteristics of the study groups.

Parameter	TTD group	PAC group	*P* value
Age, yrs	54 ± 12	54 ± 10	0.97
EuroSCORE, points	7 ± 3	7 ± 3	0.81
EuroSCORE, predicted mortality risk, %	7.5 (5.0–13.8)	10.5 (4.0–14.8)	0.65
NYHA, functional class of heart failure	3 ± 0	3 ± 1	0.19
Left ventricular ejection fraction before surgery, %	57 ± 11	57 ± 10	0.90
Duration of surgery, min	234 ± 47	229 ± 41	0.72
Duration of aortic cross-clamping, min	105 ± 31	109 ± 31	0.69
Duration of cardiopulmonary bypass, min	142 ± 43	142 ± 37	0.97

TTD: transpulmonary thermodilution; PAC: pulmonary artery catheter. Data are presented as mean ± SD or median (25th–75th percentiles).

**Table 2:** Changes in hemodynamic parameters in the study groups.

	Group	Before surgery	End of surgery	2 hrs	6 hrs	12 hrs	18 hrs	24 hrs
CVP, mm Hg	TTD group	12 ± 4	16 ± 4^†^	11 ± 4	12 ± 4*	11 ± 5	13 ± 5*	14 ± 4
	PAC group	13 ± 4	16 ± 3^†^	12 ± 4	10 ± 3^†^	10 ± 4^†^	11 ± 3^†^	12 ± 4
PAOP, mm Hg	PAC group	19 ± 7	18 ± 6	15 ± 6^†^	13 ± 4^†^	12 ± 5^†^	15 ± 6^†^	16 ± 3
GEDVI, mL m^−2^	TTD group	757 ± 191	707 ± 63	719 ± 150	747 ± 106	815 ± 203^†^	824 ± 214^†^	839 ± 205^†^
SVV, %	TTD group	8 ± 5	13 ± 5	13 ± 6	13 ± 5	14 ± 7	15 ± 5^†^	16 ± 6^†^
EVLWI, mL kg^−1^	TTD group	12 ± 4	11 ± 2^†^	10 ± 3^†^	9 ± 2^†^	10 ± 3	10 ± 3^†^	10 ± 2
MAP, mm Hg	TTD group	73 ± 15	72 ± 13*	74 ± 15	71 ± 8	80 ± 11	87 ± 16^†^	88 ± 15^†^
	PAC group	72 ± 17	66 ± 8	74 ± 11	71 ± 11	80 ± 11	87 ± 12^†^	82 ± 21
SVRI, dyne·sec^−1 ^cm^−5 ^m^−2^	TTD group	2732 ± 738	1913 ± 564^∗†^	2093 ± 711^†^	1730 ± 443^†^	1948 ± 534^∗†^	2216 ± 692^†^	2073 ± 517^†^
	PAC group	2610 ± 1039	1466 ± 411^†^	1962 ± 644^†^	2030 ± 618^†^	2345 ± 716	2493 ± 626	2286 ± 581
HR, min^−1^	TTD group	65 ± 12	82 ± 22^†^	78 ± 11^†^	74 ±11^†^	75 ± 15^†^	77 ± 14^†^	74 ± 11^†^
	PAC group	71 ± 14	79 ± 12^†^	78 ± 11^†^	79 ± 14^†^	75 ± 15	77 ± 13	80 ± 13^†^

TTD: transpulmonary thermodilution; PAC: pulmonary artery catheter; CVP: central venous pressure; PAOP: pulmonary artery occlusion pressure; GEDVI: global end-diastolic volume index; EVLWI: extravascular lung water index; MAP: mean arterial pressure; SVRI: systemic vascular resistance index; HR: heart rate.

*P < 0.05 between the groups; ^†^ *P* < 0.05 within the group compared with the preoperative value. Data are presented as mean ± SD.

**Table 3:** Changes in oxygenation and laboratory parameters in the study groups.

Parameter	Group	Before surgery	End of surgery	2 hrs	6 hrs	12 hrs	18 hrs	24 hrs
PaO_2_/FiO_2_, mm Hg	TTD group	330 ± 104	269 ± 129^†^	322 ± 124	329 ± 102	337 ± 137	311 ± 114	291 ± 82
	PAC group	279 ± 114	234 ± 89	286 ± 96	325 ± 87	324 ± 76	309 ± 101	310 ± 134
ScvO_2_, %	TTD group	73 ± 10	71 ± 15	69 ± 10	66 ± 14	69 ± 11*	65 ± 14^†^	66 ± 8^†^
	PAC group	70 ± 9	78 ± 10^†^	74 ± 10	75 ± 10	75 ± 14^†^	67 ± 14	65 ± 9
pH	TTD group	7.39 ± 0.04	7.34 ± 0.01^†^	7.35 ± 0.04^†^	7.34 ± 0.07^†^	7.35 ± 0.07^†^	7.38 ± 0.05	7.41 ± 0.05^†^
	PAC group	7.38 ± 0.05	7.34 ± 0.05^†^	7.33 ± 0.06^†^	7.38 ± 0.05	7.39 ± 0.05	7.42 ± 0.05^†^	7.43 ± 0.04^†^
Lactate, mmol L^−1^	TTD group	0.9 ± 0.3	2.8 ± 1.0^†^	2.2 ± 1.1^†^	3.3 ± 2.1^†^	3.6 ± 2.2^†^	2.5 ± 1.6^†^	2.2 ± 1.1^†^
	PAC group	0.9 ± 0.3	3.0 ± 0.9^†^	2.5 ± 1.2^†^	3.5 ± 2.1^†^	4.0 ± 2.6^†^	2.5 ± 1.2^†^	2.1 ± 0.6^†^
Hb, g dL^−1^	TTD group	12.7 ± 1.9	9.0 ± 1.4^†^	10.1 ± 2.1^†^	10.5 ± 1.7^†^	11.1 ± 1.3^†^	11.0 ± 1.5^†^	10.9 ± 1.3^†^
	PAC group	11.7 ± 1.4	8.4 ± 1.2^†^	9.6 ± 2.0^†^	10.5 ± 1.4^†^	10.7 ± 1.4^†^	10.6 ± 1.2^†^	10.6 ± 1.7^†^
Glucose, mmol L^−1^	TTD group	5.8 ± 2.0	7.3 ± 3.2	7.6 ± 3.7	11.9 ± 4.1^†^	12.0 ± 6.9^†^	8.7 ± 2.2^†^	7.8 ± 3.1
	PAC group	5.8 ± 1.7	8.5 ± 4.3^†^	8.4 ± 3.4^†^	10.3 ± 3.9^†^	12.8 ± 4.7^†^	9.3 ± 3.7^†^	8.6 ± 5.5^†^

TTD: transpulmonary thermodilution; PAC: pulmonary artery catheter; PaO_2_: partial arterial oxygen pressure; FiO_2_: fraction of inspired oxygen; ScvO_2_: central venous oxygen saturation; Hb: hemoglobin.

*P < 0.05 between the groups; ^†^ *P* < 0.05 within the group compared with the preoperative value. Data are presented as mean ± SD.

**Table 4:** Clinical characteristics of the study groups.

Characteristic	TTD group	PAC group	*P* value
Crystalloids intraoperatively, mL	1290 ± 213	1158 ± 327	0.14
Crystalloids during 24 hrs postoperatively, mL	1875 ± 531	1518 ± 410	**0.02**
Colloids during 24 hrs postoperatively, mL	250 ± 68	75 ± 41^†^	**0.04**
Incidence of colloid administration	15%	45%	0.08
Fluids during 24 hrs postoperatively, mL	1850 (1600–2575)	1550 (1312–1700)	**0.01**
Incidence of inotropic/vasopressor support	35%	65%	0.11
Duration of inotropic/vasopressor support after operation, hrs	11.9 ± 4.6	17.1 ± 3.8	0.14
Incidence of diuretic administration	30%	55%	0.20
Fluid balance at 24 hrs postoperatively, mL	85 (−358–940)	−743 (−1275–196)	0.05
Diuresis at 24 hrs postoperatively, mL	2410 ± 1196	2439 ± 959	0.93
Postoperative drainage blood loss, mL	557 ± 108	584 ± 190	0.21
SOFA score at 24 hrs postoperatively, points	5 ± 1	6 ± 1	0.37
Duration of respiratory support, hrs	14.3 ± 5.1	19.4 ± 5.8	**0.04**
Length of ICU stay, hrs	61.5 ± 37.2	64.1 ± 37.8	0.70
Length of hospital stay, days	20.7 ± 7.8	22.0 ± 7.8	0.60

TTD: transpulmonary thermodilution; PAC: pulmonary artery catheter. Data are presented as %, mean ± SD or median (25th–75th percentiles).
